# Location Detection and Tracking of Moving Targets by a 2D IR-UWB Radar System

**DOI:** 10.3390/s150306740

**Published:** 2015-03-19

**Authors:** Van-Han Nguyen, Jae-Young Pyun

**Affiliations:** 1 Department of Information and Communication Engineering, Chosun University, 375 Susuk-Dong, Dong-gu, Gwangju 501-759, Korea; E-Mail: hannvntu@gmail.com; 2 Department of Electronic and Automation Engineering, Nha Trang University, 02 Nguyen Dinh Chieu, Nha Trang 625080, Khanh Hoa, Vietnam

**Keywords:** ultra-wide band, localization, tracking, Kalman filter, extended Kalman filter, unscented Kalman filter

## Abstract

In indoor environments, the Global Positioning System (GPS) and long-range tracking radar systems are not optimal, because of signal propagation limitations in the indoor environment. In recent years, the use of ultra-wide band (UWB) technology has become a possible solution for object detection, localization and tracking in indoor environments, because of its high range resolution, compact size and low cost. This paper presents improved target detection and tracking techniques for moving objects with impulse-radio UWB (IR-UWB) radar in a short-range indoor area. This is achieved through signal-processing steps, such as clutter reduction, target detection, target localization and tracking. In this paper, we introduce a new combination consisting of our proposed signal-processing procedures. In the clutter-reduction step, a filtering method that uses a Kalman filter (KF) is proposed. Then, in the target detection step, a modification of the conventional CLEAN algorithm which is used to estimate the impulse response from observation region is applied for the advanced elimination of false alarms. Then, the output is fed into the target localization and tracking step, in which the target location and trajectory are determined and tracked by using unscented KF in two-dimensional coordinates. In each step, the proposed methods are compared to conventional methods to demonstrate the differences in performance. The experiments are carried out using actual IR-UWB radar under different scenarios. The results verify that the proposed methods can improve the probability and efficiency of target detection and tracking.

## 1. Introduction

Many applications require information about an object’s location for rescue, emergency and security purposes. The approaches that access an object’s location are typically divided into two groups: active and passive localization. In the former approach, the object is associated with a mobile station (MS), such as a tag or device in a communication network. The object’s location is determined by sharing data between the MS and the base stations (BSs) [1]. The Global Positioning System (GPS), cellular networks, Bluetooth and wireless sensor networks (WSNs) are used in active localization. In the latter approach, the object does not communicate with other devices. However, the object’s location can be determined by using the reflected signal from the object [2]. Radio detection and ranging (radar), sound navigation and ranging (sonar) and laser detection and ranging (LADAR) are the most common types of passive localization. These methods have both advantages and disadvantages. However, GPS and long-range radar generate many errors during indoor localization and tracking. Cellular networks and WSNs are limited by their complicated controls and protocols. Sonar and LADAR signals are degraded by environmental interference. Therefore, ultra-wide band (UWB) radar has become an emerging technology that is appropriate for indoor localization and tracking. UWB radar has many advantages, such as a high spatial resolution, the ability to mitigate interference, through-the-wall visibility, a simple transceiver and a low cost [3].

In this study, impulse-radio UWB (IR-UWB) radar is used to detect, localize and track a moving target in an indoor environment. IR-UWB radar has one transmitter and one receiver. The transmitter in the radar sends very narrow pulses, and the receiver receives the reflected pulses. The received signal passes through several signal-processing steps to extract the target signal. This target signal is generally perturbed by clutter, noise and attenuation. Therefore, removal of the unwanted signal and signal compensation are crucial tasks for improving the detectability of a target.

The impulse used in an IR-UWB radar has an ultra-wide bandwidth and a very weak transmission power. Since the impulse is a noise-like signal, IR-UWB signal processing is normally performed in the time domain rather than in the frequency domain. We assume that p(t) is an elementary IR-UWB waveform. The transmitted signal is:

(1) $$s\left(t\right)=\sum_{k=-\infty }^{+\infty }p\left(t-kT_{s}\right)$$

where $T_{s}$ is the pulse repetition period. Furthermore, the transmission path is from the transmitter via the target to the receiver. The response of this indoor radio channel is:

(2) $$h\left(t\right)=\sum_{n=1}^{L}{\text{α}}_{n}\text{δ}\left(t-{\text{τ}}_{n}\right)$$

where L is the number of multipath components, δ(t) is a Dirac impulse and $\alpha_{n}$ and ${\text{τ}}_{n}$ are the amplitude and propagation delay of the n -th path, respectively [4]. At the receiver, the received signal includes a reflected signal and additive noise ℵ(t), which is expressed as:

(3) $$r\left(t\right)=\sum_{k=-\infty }^{+\infty }\sum_{n-1}^{L}\alpha_{n,k}p\prime \left(t-\tau_{n,k}-kT_{s}\right)+ℵ\left(t\right)$$

where p′(t) is the “filtered” version of p(t) transferred through the channel [5]. At the receiver, the received signal is captured and sampled. Then, it is stored in frames called radar scans; each radar scan consists of n samples. A radar scan denoted as r[n] is the signal strength *versus* the sample number. These samples can be converted into a signal propagation time on the basis of the sampling rate. If we sort m continuous radar scans, they will create a radargram. Thus, a radargram is a n × m matrix in which each column is a radar scan and is denoted as $X_{n\times m}$.

**Figure 1:**
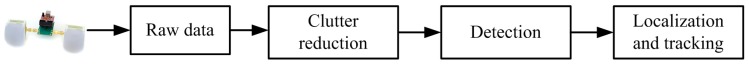
Signal processing for localizing and tracking a moving object.

For the detection, localization and tracking of a moving object, the target of interest is considered to be moving. Thus, the reflected signal from the target fluctuates according to its movement and interference from other objects. Consequently, it is convenient to represent the received signal as:

(4) $$r[n]=r_{t}[n]+r_{c}[n]+ℵ[n]$$

where $r_{t}[n]$ is the target signal reflected from the moving target and $r_{c}[n]$ is the clutter signal reflected from static objects. The signals of clutter and noise must be eliminated as much as possible from the received signal. Then, the target signature is identified using the detection step and is provided to the target localization and tracking step. The above tasks are performed via the signal-processing procedure suggested in [6] and are described in Figure 1.

Each step in the radar signal-processing procedure provides some specific functions, and its outputs are the inputs for the next step.

First, raw data are directly captured from the radar and stored for the prospective step. The raw data are often in the form of a radargram. The goal of the clutter-reduction step is to remove unwanted clutter signals as much as possible. The expected output of the clutter-reduction step is the signal reflected from only the target. The clutter reduction in IR-UWB radar for moving-target detection and tracking systems in a short-range indoor surveillance area is similar to the background-subtraction techniques in visual surveillance systems [7] and the clutter-reduction techniques in ground-penetrating radar (GPR) applications [8]. The simplest clutter-reduction method in IR-UWB radar uses a mean method that assumes that the clutter is the average of a number of previous radar scans [9]. However, its performance is poor because of its simple clutter estimation. Another mean method is exponential averaging (EA) suitable for online processing, as discussed in [10]. In this method, the clutter is estimated from the previous estimated signals and updated. A more common clutter-reduction method is based on singular value decomposition (SVD) [11,12]. This method is efficient for through-wall imaging systems using UWB radar, but it can also be used in moving-target detection, localization and tracking systems. The disadvantage of this method is that it uses a significant amount of memory and computational resources to store and compute matrices.

In the detection step, the presence or absence of a target is determined. Normally, the decision is reached on the basis of a comparison between the observed signal and the threshold. If the signal strength is greater than a certain threshold, a target is present. Otherwise, the target is absent. From the viewpoint of the threshold determination, a detection method is often divided into two groups: an optimal detector and a suboptimal detector [13]. The optimal detector is based on a statistical optimization. Because the quality of detection is determined by the probabilities of detection and a false alarm for the given target conditions, the optimal detector can provide very accurate decisions; however, its structure can be extremely complex. Therefore, a suboptimal detector is often used. To detect a moving target by IR-UWB radar, detection using a matched filter was introduced in [14]. The precision of this method depends on how well the received signal and template match. In an IR-UWB radar application, the pulse width is very narrow, and the pulse waveform is strongly affected by the target distance, material and shape. Therefore, the matching ratio between a reflected signal and a template pulse is small. As another example, constant false alarm rate (CFAR) detection was proposed in [15]. The greatest difficulty in this method is determining the noise and clutter distributions in order to define a suitable threshold. In [16], detection was carried out by the CLEAN algorithm, which searches all pulse presences by the cross-correlation between the received signal and the template signal and then compares them to a threshold. However, the received signal strength will be weaker when the target distance is greater. Therefore, it is not appropriate to interpret all of the received signal strengths under the same conditions.

The last step, localization and tracking, involves the association of consecutive observations of the same target with its location and track. In general, the target location and track in a localization and tracking system via UWB radios can be determined on the basis of the angle of arrival (AOA), the received signal strength (RSS), the time difference of arrival (TDOA) and the time of arrival (TOA) [17]. However, an IR-UWB radar can only provide the TOA from the target. In other words, the radar observes the target TOA. The target TOA can easily be converted to the target distance by multiplying the TOA by the speed of light (*i.e.*, d=c×TOA , where d is the target distance and $c=3\times 10^{8}$ m/s is the speed of light). Several methods have been investigated to solve the problem of target localization and tracking with UWB radar. In [18,19,20,21], target localization using a multistatic radar system was proposed. For this application, the multistatic radar system consists of one transmitter and two or more receivers. The distance (or TOA) observed from each receiver creates a circle in x and y coordinates. The intersection of these circles is the location of the target. However, because of noise, these circles usually intersect in several places that are possible target areas. To estimate the target location from the possible target area, a number of methods were proposed, including the least-squares method, the spherical-interpolation method, the Taylor-series method and a two-stage method that combines a linear Kalman filter (KF) and the Taylor-series method [22,23]. In [24], an expanded multistatic UWB radar system was proposed. In this case, two independent multistatic radars cooperate to localize and track single and multiple targets. A method of joining the intersections of ellipses was introduced. This method is based on a geometric interpolation of target localization; the target position is estimated by using a properly created cluster of ellipse intersections that represent potential target positions. Another solution for target tracking in a UWB radar network was described in [25,26,27,28]. In this solution, the radar network consists of many nodes, which provide sufficient observations for estimating the target tracks by a particle filter. The proposed approach provides high accuracy, even at low signal-to-noise ratios, with both static and dynamic clutter, and it can track complicated maneuvering trajectories.

In this paper, a new combination consisting of signal-processing procedures is proposed for the tracking of a moving target. In addition, advanced algorithms are introduced for each signal-processing step. In the clutter-reduction step, a KF method for estimating clutter is presented and compared to existing methods. In the detection step, the conventional CLEAN detection algorithm is modified. We overcome the problems of the conventional CLEAN detection algorithm by compensating for the weak signal and adding a window method. Comparisons of the clutter-reduction and detection methods performed with one radar senor were partially presented in [29,30]. In the next processing step, localization and tracking, we introduce different approaches using an extended KF (EKF) and unscented KF (UKF) to estimate the target trajectory. In this paper, these steps are concatenated in order and implemented with two radars to determine the coordinates of the moving target.

## 2. Signal Processing Steps for Moving-Target Detection, Localization and Tracking Using IR-UWB Radar

### 2.1. Clutter Reduction

In Equation (4), the received signal is divided into three parts: the target signal, clutter and noise. To reduce the clutter, the simplest solution is to estimate the clutter and subtract it from the received signal. In this paper, we introduce a clutter-reduction method based on a KF and compare its performance with existing methods, such as EA- and SVD-based clutter reduction.

#### 2.1.1. Exponential Averaging Clutter-Reduction Method

In [10], a clutter-reduction method was proposed by applying exponential averaging. In this method, the raw data to be processed are radar scans. Given an initial estimated clutter signal $r_{c(k-1)}^{\sim }$, the new estimated clutter signal $r_{c(k)}^{\sim }$ is computed recursively from $r_{c(k-1)}^{\sim }$ and the new incoming radar scan $r_{k}$, where *k* is the time index. Thus, $r_{c(k)}^{\sim }$ is derived as:

(5) $$r_{c\left(k\right)}^{\sim }=\text{α}r_{c\left(k-1\right)}^{\sim }+\left(1-\text{α}\right)r_{k}\;=r_{c\left(k-1\right)}^{\sim }+\left(1-\text{α}\right)\left(r_{k}-r_{c\left(k-1\right)}^{\sim }\right)\;=r_{c\left(k-1\right)}^{\sim }+\left(1-\text{α}\right)s_{k}$$

where α is a constant scalar weighting factor and $s_{k}=r_{k}-r_{c(k-1)}^{\sim }$ is a one-dimensional (1D) vector with the same size as a radar scan. Thus, the new estimated clutter consists of a fraction of the previous estimate and a fraction of the current radar scan. The weighting factor α is an empirical scalar that takes values between zero and one. It controls the amount of averaging in the estimated clutter. $s_{k}$ is the result of subtracting the previous estimated clutter from the current incoming radar scan, and it is considered the target signal.

#### 2.1.2. Singular Value Decomposition Clutter-Reduction Method

In through-the-wall imaging systems that use UWB radar, a clutter-reduction technique based on SVD is often used [11,12]. However, this method can also be applied to moving-target detection, localization and tracking systems that use IR-UWB radar. SVD is a matrix factorization technique. The main aim of SVD is to split the scan matrix into subspaces that correspond to the clutter, target and noise so that the clutter can then be rejected. The raw data used in this method must be a radargram $X_{n\times m}$. The SVD of the matrix $X_{n\times m}$ is given by:

(6) $$X_{n\times m}=USV^{T}$$

where U and V are n×n and m×m unitary matrices, respectively. $V^{T}$ is the transposed matrix of V. S is an n×m diagonal matrix containing the square roots of the eigenvalues from U and V in descending order, *i.e.*, $S=diag\left({\text{σ}}_{1},{\text{σ}}_{2},\cdots ,{\text{σ}}_{r}\right)$, with ${\text{σ}}_{1}\geq {\text{σ}}_{2}\geq ...\geq {\text{σ}}_{r}$. The SVD of the matrix $X_{n\times m}$ can be alternatively represented by “rank-one decomposition,” as follows:

(7) $$X_{n\times m}=USV^{T}={\text{σ}}_{1}\left(\begin{array}{l} ...\\ u_{1}\\ ... \end{array}\right)\left(...v_{1}^{T}...\right)+...+{\text{σ}}_{n}\left(\begin{array}{l} ...\\ u_{n}\\ ... \end{array}\right)(...v_{n}^{T}...)\;=\sum_{i=1}^{m}{\text{σ}}_{i}u_{i}v_{i}^{T}=M_{1}+M_{2}+...+M_{m}=\sum_{i=1}^{m}M_{i}$$

where $M_{i}$ are matrices of the same dimension as X and are called modes or the *i*-th eigenimage. Subsequently, the radargram X is split into three parts: the target signal matrix $M_{t}$, clutter matrix $M_{c}$ and noise matrix $M_{n}$, expressed by:

(8) $$X_{n\times m}=M_{t}+M_{c}+M_{n}$$

If we assume that the clutter signal strength is higher than the target signal and noise, $M_{1}$ represents the clutter, $M_{2}$ represents the target signal and the rest is noise.

#### 2.1.3. Proposed KF-Based Clutter-Reduction Method

In estimation theory, a KF provides an optimal-state estimation technique for linear dynamic systems [31]. Assuming that the system is a discrete system, the general state-estimation problem is stated as follows. We suppose the state $x_{k}$ of a dynamic system is governed by the state transition equation given by:

(9) $$x_{k}=f\left(x_{k-1},u_{k-1},w_{k-1}\right)$$

where $u_{k-1}$ is the control input, $w_{k}$ is process noise and k is the time index. Because of noise, the state is hidden. We only observe the measurement of the state, which is related to the state by the following measurement equation:

(10) $$z_{k}=g\left(x_{k},v_{k}\right)$$

where $z_{k}$ is the measurement of the state and $v_{k}$ is measurement noise. Our purpose is to estimate the system state on the basis of the modeling of the system process and measurement, as in Equations (9) and (10).

If f(.) and g(.) are linear functions and *w* and *v* are Gaussian additive noise with the covariance matrices Q and R, respectively, the state transition Equation (9) and measurement Equation (10) can then be rewritten as:

(11) $$x_{k}=Ax_{k-1}+Bu_{k-1}+w_{k-1}\;z_{k}=Hx_{k}+v_{k}$$

where A, B and H are the state-transition, control-input and measurement matrices, respectively. In this case, the state is estimated optimally using the KF algorithm. The KF algorithm, working recursively, has two steps: a time update and measurement update, which are given by the following equations:

- Time update:
  (1) Initial state and error covariance: $x_{k-1}^{\sim }$, $P_{k-1}$.
  (2) Project the state ahead: xk^=Axk−1~+Buk−1.
  (3) Project the error covariance ahead: Pk^=APk−1AT+Q.
- Measurement update:
  (1) Compute the Kalman gain: Kk=Pk^HT(HPk^HT+R)−1.
  (2) Update the estimation with the measurement: xk~=xk^+Kk(zk−Hxk^).
  (3) Update the error covariance: Pk=(I−KkH)Pk^.

For clutter reduction, a KF is used to estimate the clutter, which consists of *n* samples of a radar scan, *i.e.*, $x_{k}^{\sim }=r_{c(k)}$. Hence, the KF estimates n points of clutter independently. The measurements are the raw data in the form of radar scans, *i.e.*, $z_{k}=r_{k}$. Because the clutter is the reflection from static objects, it is considered to be constant in time. Therefore, the values assigned to the matrices are as follows: A=I, B=0 and H=I, where I is an identity matrix. The KF equations for clutter reduction are reduced to the following:

- Time update:
  (1) Initial state and error covariance: $x_{k-1}^{\sim }$, $P_{k-1}$.
  (2) Project the state ahead: xk^=xk−1~.
  (3) Project the error covariance ahead: Pk^=Pk−1+Q.
- Measurement update:
  (1) Compute the Kalman gain: Kk=Pk^(Pk^+R)−1.
  (2) Update the estimate with the measurement: xk~=xk^+Kk(zk−xk^).
  (3) Update the error covariance: Pk=(I−Kk)Pk^.

Finally, the estimated clutter is subtracted from the received radar scan in order to obtain the target signal.

### 2.2. Detection

In the detection step, we must search the reflected pulses from the target to determine whether or not a target is present. The most popular method is to compare the reflected signal against a certain threshold. The target is determined to be present whenever the reflected signal strength is greater than the threshold. Otherwise, the target is absent. Therefore, the design of an appropriate threshold is very important. In this study, we propose a modified CLEAN detection algorithm that firstly compensates for the weak signal transferred from the faraway target and secondly adds the jumping-window method to improve the probability of target detection.

#### 2.2.1. CLEAN Detection Algorithm

**Figure 2:**
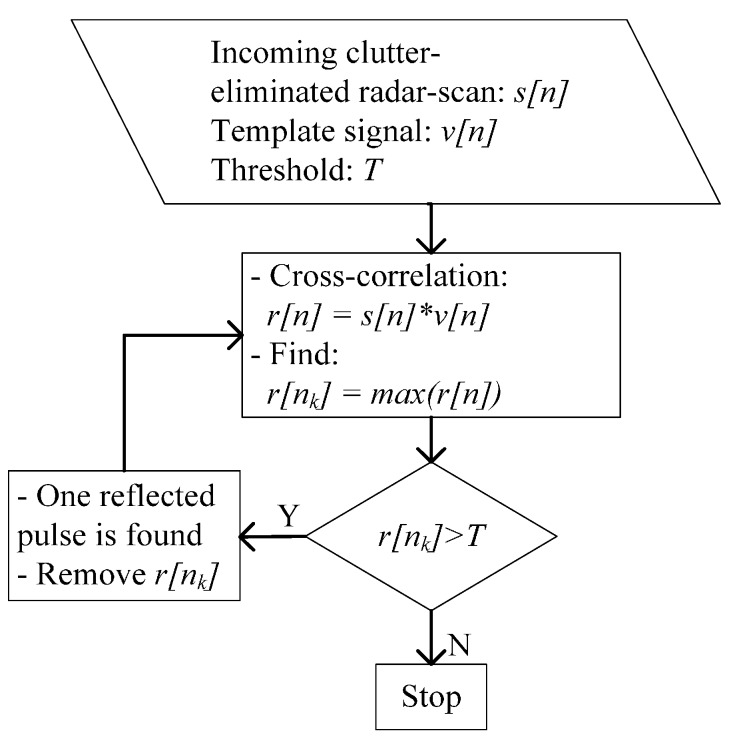
CLEAN detection algorithm.

In [16], a detection method—the CLEAN algorithm—was proposed. The inputs of the CLEAN detection method are the clutter-eliminated radar scan s[n], template signal v[n] and predefined threshold T. In this study, the template signal is observed as the signal reflected from a metal plate placed 1 m from the radar. The conventional CLEAN algorithm uses a fixed threshold for all radar scans. T is determined by the average energy in a radar scan multiplied by a scalar. Figure 2 shows a cycle of the CLEAN algorithm for one radar scan. As shown in Figure 2, the CLEAN algorithm searches for the reflected signals from targets on the basis of a comparison of the cross-correlation results to the threshold. First, s[n] is cross-correlated with v[n]. Second, the maximum amplitude of the cross-correlation result is compared to the threshold.

If the maximum value, $v[n_{k}]$, is greater than the threshold, one sample of a reflected pulse will be considered to be found. Then, another iteration is performed from the cross-correlation step until the maximum of the cross-correlation result is lower than the threshold. Then, all reflected pulses are found, and the iteration process will stop.

#### 2.2.2. Modified CLEAN Detection Algorithm

When an electromagnetic wave propagates in a wireless channel, its power density is attenuated. This phenomenon is known as path loss (PL). PL is defined as the ratio of the received signal power $P_{rx}$ to the transmitted signal power $P_{tx}$. In UWB wireless systems, PL is dependent on the frequency of the UWB signal and the distance from the radar to the target. For simplicity, the distance and frequency dependencies can be treated independently, as follows:

(12) $$PL\left(f,d\right)=\frac{P_{rx}}{P_{tx}}=PL\left(f\right)PL\left(d\right)$$

where $PL(f)\propto f^{-2\kappa }$ and $PL(d)\propto d^{-n}$, with κ and n denoting the frequency decay factor and the PL exponent, respectively. In an indoor environment, the frequency decay factor was observed to be 1.7 and 3.5–4.1 for line of sight (LOS) and non-line of sight (NLOS) propagation [32]. In other words, the amplitude of the received signal is inversely proportional to the distance.

In signal attenuation, a target that is further away results in a weaker reflected signal. To generate an equal condition for the signal strength, the weak signal should be compensated before proceeding to the detection determination step. To compensate for a weak signal, it is simply multiplied by a vector containing weighting factors. The weaker part of the signal must be multiplied by a higher scalar in the weight and *vice versa*. Thus, the final compensated signal is:

(13) s′[n]=s[n]α[n]

where s[n] is the signal before compensation and α[n] is a vector containing weighting factors. In our experiments, we determined that the vector containing weighting factors is proportional to the distance. After that, the conventional CLEAN algorithm was applied to the compensated signal.

**Figure 3:**
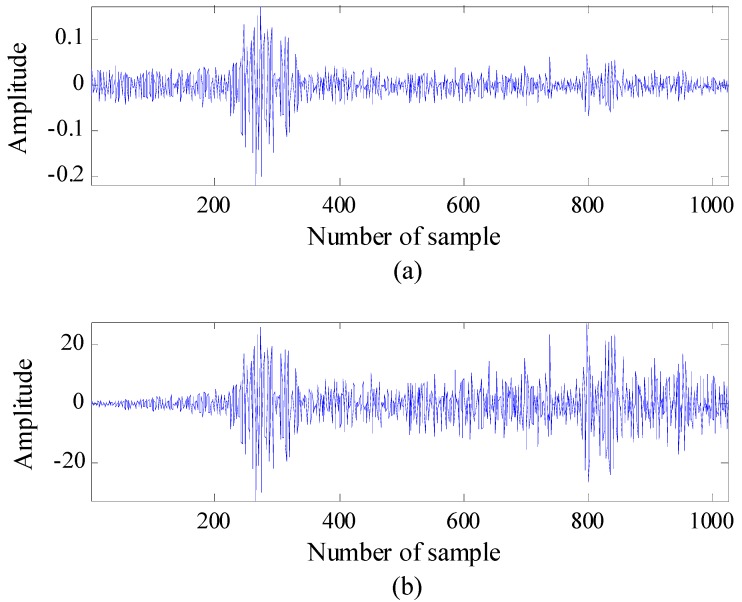
Compensation for a weak signal (two targets are moving in this example): (**a**) the signal observed before compensation and (**b**) the signal observed after compensation.

Figure 3 shows the observed signal before and after compensation, respectively. It can be seen that the reflected signal strengths of both nearby and faraway targets are approximately the same. Thus, the threshold is fairly applied for both types of targets.

After applying the CLEAN algorithm to the compensated signal, the detectability of the faraway located target increases. However, the compensation may cause another false alarm; the target signal is amplified by multiplying it with the vector containing weighting factors, and the noise may be highly amplified. In particular, the noise generated at the far field can be multiplied by a larger weighting factor. The noise may exceed the target signal strength and become a false alarm. In order to reduce the number of false alarms, the threshold must be adaptively chosen according to the range to the target. In this study, we introduce a different criterion to eliminate false alarms.

**Figure 4:**
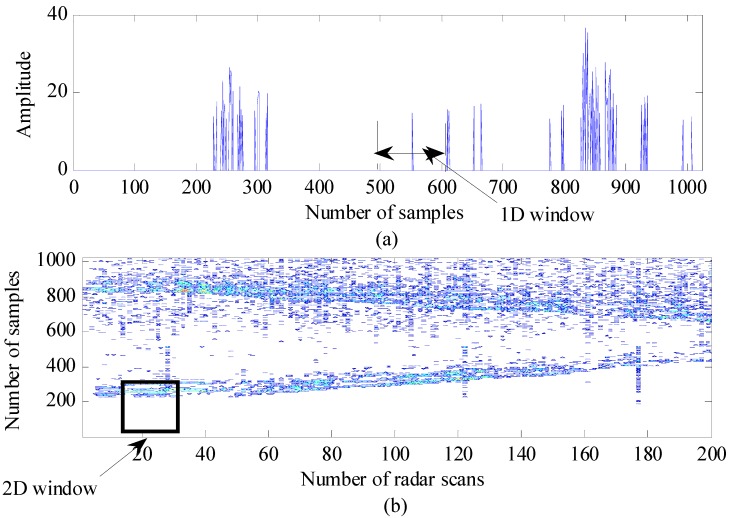
Jumping-window method for eliminating false alarms: (**a**) one-dimensional (1D) window and (**b**) two-dimensional (2D) window.

As stated in Section 2.1, the signals reflected from the target have multipath components. Because some of the multipath components may belong to one target, they can be located close together in the radar scan. Figure 4a shows the signal strength after applying CLEAN detection to the compensated signal, where two targets are located at approximately 1 m and 3 m in front of the radar. The target signal consists of multiple pulses that are reflected from different parts of the target; thus, they appear near each other. However, false alarms are present at various locations in the radar scan. Because of their presence, a jumping window is applied to eliminate false alarms, described as follows. The window size covers the appearance of targets in the radar scan and jumps along the radar scan. Then, the number of nonzero samples inside the window is examined. If it exceeds a predefined threshold, a target is present; otherwise, it is a false presence, and the nonzero samples inside the window are deleted. This method is called the 1D jumping window.

Although the 1D jumping window method can eliminate a majority of false alarms, the alarms often appear close together within a certain range. Therefore, the 1D jumping window method cannot provide results of sufficient quality. In this case, we extended the 1D jumping window method using the properties of the neighbors in the currently examined radar scan. Normally, the movement of a target is not as fast as that for an indoor application, and the radar scan rate is considerably high (e.g., 24 radar scans per second). Therefore, there is a slight difference in the target location between two consecutive radar scans. Figure 4b presents a radargram consisting of 200 continuous radar scans after CLEAN detection. In this figure, the dot patterns indicate both detected targets and false alarms. However, the dot patterns of the detected targets appear close together and generate trends over a number of radar scans. Thus, the number of dot patterns in a detected-target area is greater than the number of dot patterns in a false-alarm area. By taking this into account, the extension of the 1D jumping window is described as follows. A 2D window, in which the number of columns m is related to the number of radar scans and the number of rows *n* is related to the number of samples, is proposed. The window jumps along and between the radar scans. Similar to the 1D jumping window, the total number of nonzero samples inside the window is compared to a threshold in order to determine if the target is present or absent. The target is considered to be present if the number of nonzero samples inside the window is higher than the threshold and *vice versa*. This method is called the 2D jumping window. In this work, we set the window size to be quite small at 10 samples × 10 radar scans and count the number of samples indicating the target.

### 2.3. Localization and Tracking

Localization and tracking are required to consecutively observe the location and track of the target. In our application, two radars are used to observe the target. We assume that the target location to be determined is (dx,dy), and the positions of the radars are $\left(X_{i},Y_{i}\right)$, where i=1,  2 is the radar number. The radar observations generate two circles with radii $r_{1}$ and $r_{2}$, which are the distances from the radars to the target. Thus, the target location is determined by the intersection between the two circles:

(14) $$\left\{ \begin{array}{l} {\left(dx-X_{1}\right)}^{2}+{\left(dy-Y_{1}\right)}^{2}=r_{1}^{2}\\ {\left(dx-X_{2}\right)}^{2}+{\left(dy-Y_{2}\right)}^{2}=r_{2}^{2} \end{array}\right.$$

However, because of noise, the intersection created by the two circles does not represent the target location exactly. Therefore, specific signal processing is required to estimate the true target location from the noisy observations. In this study, we introduce two estimation methods based on the EKF and UKF methods and compare them for the better choice.

#### 2.3.1. Extended KF Localization and Tracking

We recall the general estimation problem in Equations (9) and (10), as stated in Section 2:

(15) $$\begin{array}{l} x_{k}=f\left(x_{k-1},u_{k-1}\right)+w_{k-1}\\ z_{k}=g\left(x_{k}\right)+v_{k} \end{array}$$

If $x_{k}$ or $z_{k}$ is a nonlinear function, the estimation of the KF should be linearized by using approximation techniques. A KF with linearization is called an EKF. The most common approximation technique used in the EKF is the Taylor series [33,34].

The moving-target state is characterized by its location and velocity in *x* and *y* coordinates, *i.e.*, $x_{k}={\left[dx,vx,dy,vy\right]}^{T}$. The state transition is governed by the following motion equations:

(16) $$x_{k}=\left[\begin{array}{c} dx_{k}\\ vx_{k}\\ dy_{k}\\ vy_{k} \end{array}\right]=f(x_{k-1},u_{k-1})+w_{k-1}=\left[\begin{array}{c} dx_{k-1}+vx_{k-1}t+a_{x}\frac{t^{2}}{2}\\ vx_{k-1}+a_{x}t\\ dy_{k-1}+vy_{k-1}+a_{y}\frac{t^{2}}{2}\\ vy_{k-1}+a_{y}t \end{array}\right]+w_{k-1}=Ax_{k-1}+Bu_{k-1}+w_{k-1}$$

where *dx*, *dy*, *vx* and *vy* are the positions and velocities of the target in x and y coordinates.

The state transition matrix is expressed as $A=\left[\begin{array}{cccc} 1 & t & 0 & 0\\ 0 & 1 & 0 & 0\\ 0 & 0 & 1 & t\\ 0 & 0 & 0 & 1 \end{array}\right]$, whereas the control input matrix is described as $B=\left[\begin{array}{cccc} \frac{t^{2}}{2} & 0 & 0 & 0\\ 0 & t & 0 & 0\\ 0 & 0 & \frac{t^{2}}{2} & 0\\ 0 & 0 & 0 & t \end{array}\right]$. In addition, the input vector is $u_{k}={\left[a_{x},a_{x},a_{y},a_{y}\right]}^{T}$, where $a_{x}$ and $a_{y}$ are the target accelerations in x and y coordinates, respectively. In addition, w=ℵ(0,Q) is the additive process noise with the covariance matrix Q.

The radars can measure only the distance from the target to the radars, *i.e.*, $z_{k}={\left[r_{1},r_{2}\right]}^{T}$. Thus, the relationship between the target distances measured by the radars and the target position is given by the measurement equation:

(17) $$z_{k}=\left[\begin{array}{c} r_{1}\\ r_{2} \end{array}\right]=g(x_{k})+v_{k}=\left[\begin{array}{c} \sqrt{{\left(dx-X_{1}\right)}^{2}+{\left(dy-Y_{1}\right)}^{2}}\\ \sqrt{{\left(dx-X_{2}\right)}^{2}+{\left(dy-Y_{2}\right)}^{2}} \end{array}\right]+v_{k}$$

where $v_{k}=ℵ(0,R)$ is the additive measurement noise with the covariance matrix R. It can be seen that the measurement equation is nonlinear. Therefore, the EKF algorithm must be applied to estimate the target state. Similar to the conventional KF algorithm, the EKF algorithm contains a time-update step and a measurement-update step, as follows:

- Time update:
  (1) Initial state and error covariance: $x_{k-1}^{\sim }$, $P_{k-1}$.
  (2) Project the state ahead: xk^=Axk−1~+Buk−1.
  (3) Project the error covariance ahead: Pk^=APk−1AT+Q.
- Measurement update:
  (1) Compute the measurement Jacobian matrix:
    (18) $$H_{k}=\left[\begin{array}{c} \frac{\partial g_{1}}{\partial x_{k}}\\ \frac{\partial g_{2}}{\partial x_{k}} \end{array}\right]=\left[\begin{array}{c} \frac{dx_{k}-X_{1}}{\sqrt{{\left(dx_{k}-X_{1}\right)}^{2}+\left(dy_{k}-Y_{1}\right)}},0,\frac{dy_{k}-Y_{1}}{\sqrt{{\left(dx_{k}-X_{1}\right)}^{2}+\left(dy_{k}-Y_{1}\right)}},0\\ \frac{dx_{k}-X_{2}}{\sqrt{{\left(dx_{k}-X_{2}\right)}^{2}+\left(dy_{k}-Y_{2}\right)}},0,\frac{dx_{k}-Y_{2}}{\sqrt{{\left(dx_{k}-X_{2}\right)}^{2}+\left(dy_{k}-Y_{2}\right)}},0 \end{array}\right]$$
  (2) Compute the Kalman gain: Kk=Pk^HkT(HkPk^HkT+R)−1.
  (3) Update the estimate with the measurement: xk~=xk^+Kk(zk−g(xk^)),
    where $g\left(x\right)=\sqrt{{\left(dx-X_{i}\right)}^{2}+{\left(dy-Y_{1}\right)}^{2}}\text{, }i=1,\;2$.
  (4) Update the error covariance: Pk=(I−KkHk)Pk^.

#### 2.3.2. Unscented KF Localization and Tracking

Although the EKF has been used widely for nonlinear state estimation, the EKF is sometimes difficult to tune and implement. The EKF is only reliable for systems that are almost nonlinear. The EKF uses linearization techniques based on the Taylor-series approximation, in which high-order derivations are ignored. Thus, it may cause a high error. To overcome this problem, a different approach for linearization is introduced: the UKF [35,36]. The main difference between the UKF and the EKF is the linearization method. Although the EKF uses a Taylor series to calculate the mean and covariance of the state distribution, the UKF uses an unscented transformation (UT), in which a set of statistical points (sigma points) that propagate through nonlinear functions are used to parameterize the mean and covariance of the state distribution.

The application of the UKF to target localization and tracking using IR-UWB radar is described as follows. First, we generate sigma points according to the following steps:

(1) Define three parameters to calculate the weight vector: α=1,β=2,κ=0.
(2) Define the size of the state vector: $L=n_{x}$.
(3) Calculate the weight vector: $\text{λ}={\text{α}}^{2}\left(L+\kappa \right)-L$ $\begin{array}{l} W_{0}^{m}=\text{λ}/\left(L+\text{λ}\right)\\ W_{0}^{c}=\text{λ}/\left(L+\text{λ}\right)+1-{\text{α}}^{2}+\text{β}\\ W_{i}^{c}=W_{i}^{m}=1/\left[2\left(L+\text{λ}\right)\right],\;i=1,\;2\;,....,\;2L \end{array}$.
(4) Initial state and covariance: $x_{k-1}^{\sim },P_{k-1}$.
(5) Calculate the sigma points: ${\text{χ}}_{k-1}=[x_{k-1}^{\sim },x_{k-1}^{\sim }+\sqrt{L+\text{λ}}\sqrt{P_{k-1}},x_{k-1}^{\sim }-\sqrt{L+\text{λ}}\sqrt{P_{k-1}}]$.

Second, the sigma points are propagated through the state and measurement equations to calculate the mean and covariance of the state distribution. Similar to the KF, the UKF, operating recursively, consists of a time update and measurement update, as follows:

- Time update
  (1) Propagate each sigma point through the state equation: ${\text{χ}}_{k}^{\left(i\right)}=A{\text{χ}}_{k-1}^{\left(i\right)}+Bu_{k-1}\text{, }i=0,\;1,...,\;2L$.
  (2) Project the state ahead: xk^=Axk−1~+Buk−1.
  (3) Project the error covariance ahead: Pk^=APk−1AT+Q.
- Measurement update
  (1) Propagate each sigma point through the measurement equation: $\psi_{k}^{\left(i\right)}=g(\chi_{k-1}^{\left(i\right)})\text{, }i=0,\;1,\;...,\;2L$, where $g\left(x\right)=\sqrt{{\left(dx-X_{i}\right)}^{2}+{\left(dy-Y_{i}\right)}^{2}}\text{, }i=1,\;2$.
  (2) Predict the measurement: zk^=∑i=02LWimψk(i).
  (3) Calculate the auto-covariance of the predicted measurement: Pkzz=Rk+∑i=02LWic(ψk(i)−zk^)(ψk(i)−zk^)T.
  (4) Calculate the cross-covariance of the state and predicted measurements:
    Pkxz=∑i=02LWic(ψk(i)−xk^)(ψk(i)−zk^)T.
  (5) Calculate the Kalman gain: $K_{k}=P_{k}^{xz}{\left(P_{k}^{zz}\right)}^{-1}$.
  (6) Update the state estimate with the measurement: xk~=xk^+Kk(zk−zk^).
  (7) Update the error covariance: Pk=Pk^+KkPkzzKkT.

## 3. Experimental Results

**Figure 5:**
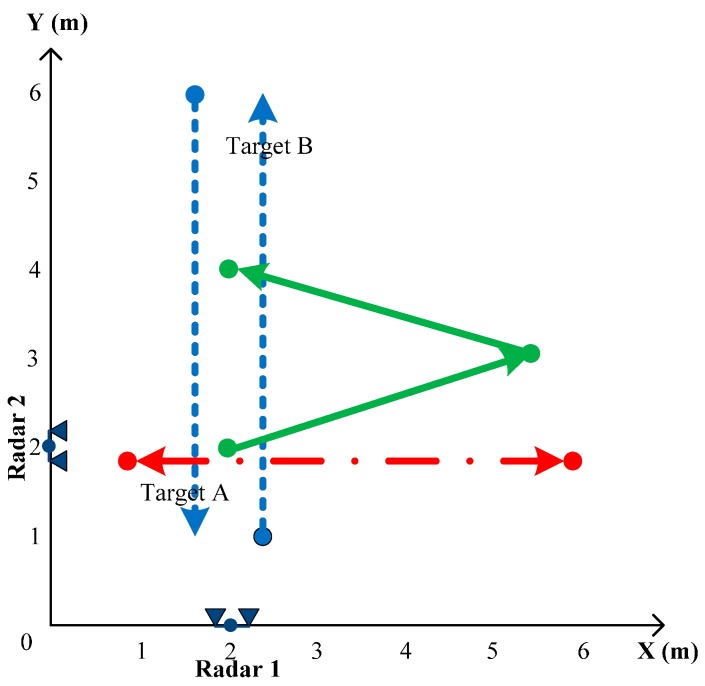
Locations of radars and the directions of target movement in the experiments.

To demonstrate signal processing for moving-object detection, localization and tracking using IR-UWB radar, several experiments were carried out. The experiments were performed in a classroom that contained a whiteboard, tables and chairs. The target was represented by one or two people moving within the range of the radar. The IR-UWB radars used in the experiments were equipped with NVA 6100 chipsets produced by Novelda and Vivaldi directional antennas with an opening angle of 20° (V) × 50° (H) [37]. The performance conditions of the developed IR-UWB radar are shown in Table 1. The radars were set up to work at a pulse repetition frequency (PRF) of 48 MHz and a radar scan rate of approximately 24 scans per second. Under the condition of this PRF and averaging technique of frames, a frame can be expected to measure the 2-m distance between radar and target. The frame could be repositioned to measure beyond a 2-m distance, and thus, the radar could include the reflections from the reflecting objects located even in an 8-m away location. Furthermore, two radars are located at the classroom of 8 m × 8 m, as shown in Figure 5. The locations of two radars were determined based on the coverage limit of radar antennas. The transmitted pulse width was approximately 0.7 ns. This yielded a frequency range of 3.1 GHz to 5.6 GHz. For convenience in evaluating the performance of different signal-processing algorithms, various experimental scenarios were created, as shown in Figure 5.

**Table 1:** Operation conditions of the experimental impulse radio ultra-wide band (IR-UWB) radar.

Conditions	Value
Pulse width	0.7 ns
Number of sample in a frame	1024
Pulse repetition frequency (PRF)	48 MHz
Frame range	Approximately 2 m (in 48 MHz·PRF)

The first experiment illustrates the performance of the clutter-reduction techniques. One person moves within the range of Radar 2 from 1 m to 6 m along the path indicated by the dash-dot line in Figure 5. The signals are captured by the IR-UWB radar and are processed with three different clutter-reduction techniques: the EA, SVD and KF-based methods. The experiment is repeated without the moving target. Thus, the signals captured by the radar show the true clutter. This will be compared to the estimated clutter in order to evaluate the performance of the clutter-reduction techniques.

The second experiment is carried out to study detection. We use Radar 1 to observe two people moving on the path indicated by the dotted lines in Figure 5. The first person (Target A) moves from 6 m to 1 m, whereas the second person (Target B) moves in an opposite trajectory from 1 m to 6 m. The received signals are processed by the KF-based clutter-reduction method. After that, we apply conventional CLEAN detection and our modified CLEAN detection to the clutter-eliminated data. The parameters for these methods are listed in Table 2. In our work, the 2D window size and thresholds are chosen on the basis of our empirical adjustment.

**Table 2:** Parameters used in the detection signal processing. EA, exponential averaging.

Parameters	Value
Exponential factor in EA method	α = 0.95
Compensated vector in modified CLEAN algorithm	α[*n*] = [1,2,...1024] for *n* = 1, 2, ..., 1024
Threshold in modified CLEAN algorithm	T = 3 × mean of compensated-radar-scan signals
2D window size	10 samples × 10 radar scans

The third experiment is carried out for tracking the moving target. We use Radar 1 and Radar 2 cooperatively. In this scenario, two radars are placed orthogonally at (2 m, 0 m) and (0 m, 2 m). One person moves according to the predefined path indicated by the solid line in Figure 5. The observed data are processed by the KF-based clutter-reduction method and modified CLEAN detection algorithm before proceeding to the tracking step. We apply both EKF and UKF tracking techniques to compare their performance.

**Figure 6:**
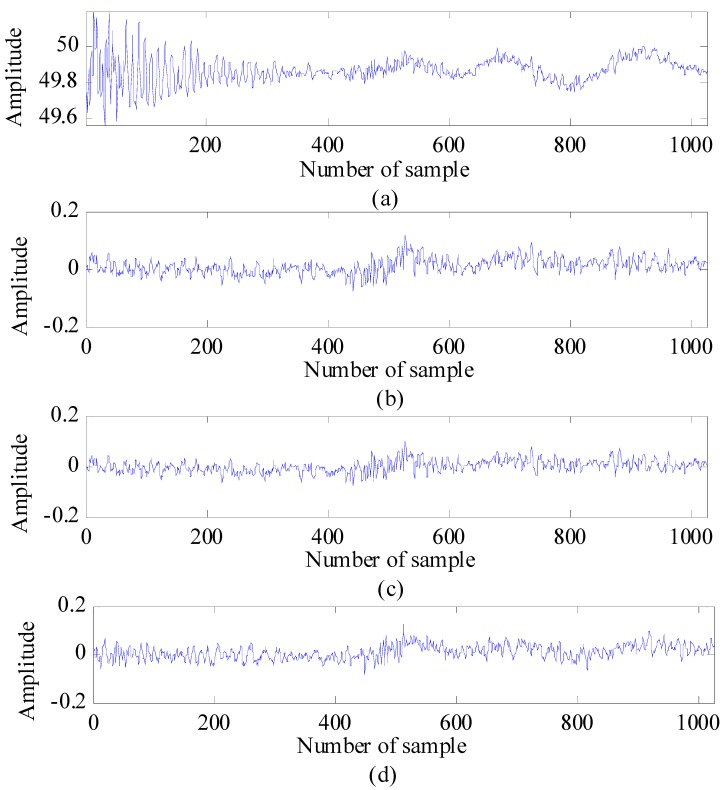
Radar scan: (**a**) before clutter reduction; (**b**) after application of clutter reduction, (**c**) after application of SVD clutter reduction; and (**d**) after application of KF-based clutter reduction.

**Figure 7:**
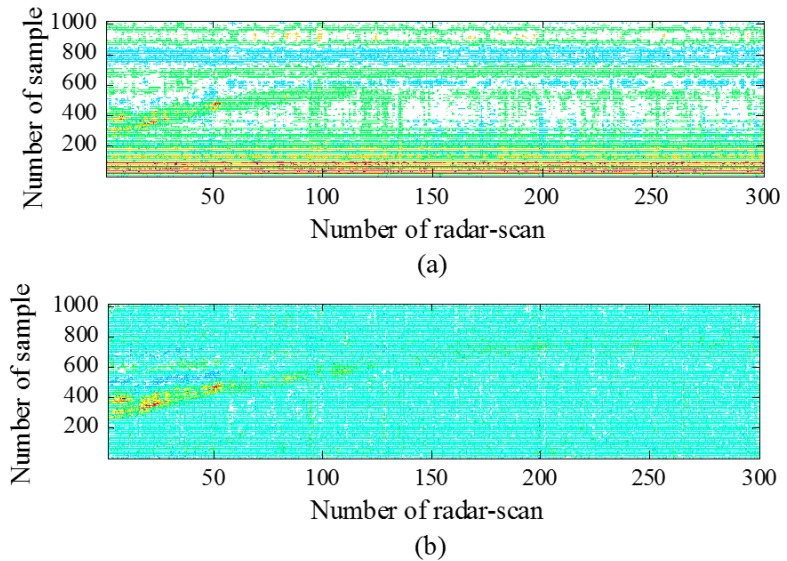

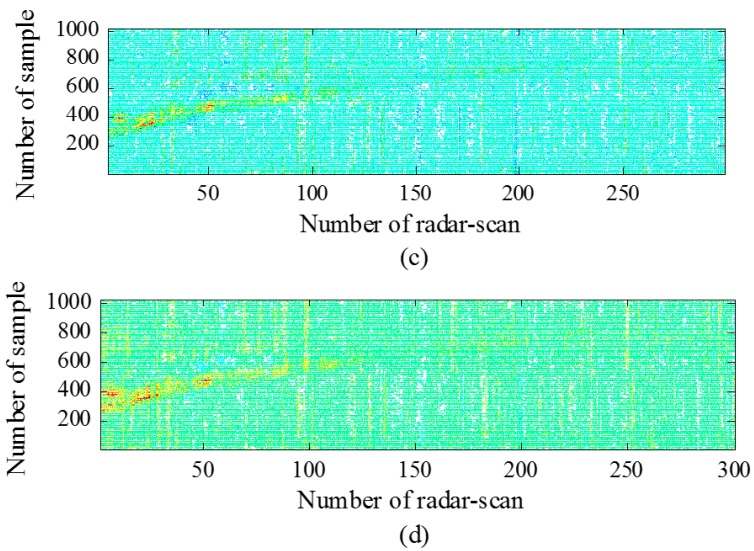
Radargrams: (**a**) before clutter reduction; (**b**) after application of EA clutter reduction; (**c**) after application of SVD clutter reduction; and (**d**) after application of KF-based clutter reduction.

Now, the obtained results of each signal-processing step will be analyzed and discussed in detail. The results for clutter reduction are shown as the observed signal before and after the application of clutter reduction and the average root mean square error (a−RMSE) between the estimated clutter and the true clutter (measured as a reference). Figure 6 shows a radar scan before and after the application of the three different clutter-reduction techniques. In this case, the target is indicated as the reflected pulse located around sample Number 550. Figure 7 shows the radargrams before and after the application of the three clutter-reduction methods. In this figure, target movement can be observed. In general, it can be observed from both Figure 6 and Figure 7 that it is difficult to recognize the target signal from the received signal before the application of clutter reduction, because the clutter affects the target signal. However, the target signal appears clearly after the clutter is removed. The comparison results of a−RMSE for each method are summarized in Table 3. The definition of a−RMSE is as follows:

(19) a−RMSE=1M∑i=1M(1N∑j=1N(rc(i,j)−rc(i,j)^)2)

where M is the number of radar scans and N is the number of samples. From Table 3, the KF-based clutter-reduction method exhibits better performance for estimating the clutter compared to the other methods.

**Table 3:** Performance comparison of different clutter-reduction methods.

Clutter-Reduction Method	Average RMSE
Kalman Filter	0.1029
Exponential Average	0.2031
Singular Value Decomposition	0.1342

**Figure 8:**
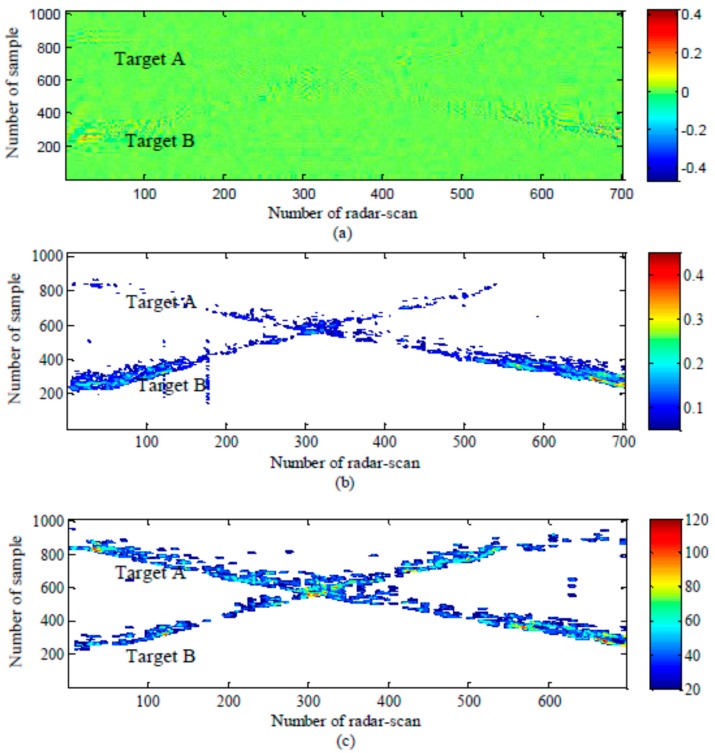
Radargrams: (**a**) before detection; (**b**) after detection with the conventional CLEAN algorithm; and (**c**) after detection with the modified CLEAN algorithm.

The results for the detection step are presented in Figure 8 and Table 4. Figure 8 shows radargrams that represent a target’s movement before and after the detection step. It can be seen that targets located close to sample Numbers 200~600 appear clearly. They are effectively detected by both the conventional CLEAN and modified CLEAN detection methods. However, targets located faraway, from around sample Number 600 to sample Number 1024, are missed when the conventional CLEAN detection method is applied. However, the modified CLEAN detection method detects the target well in most of the locations, because of the proposed signal compensation and 2D jumping window-based false alarm elimination.

Next, the results are analyzed further to compute the detection rate. The detection rate is the ratio between the number of radar scans in which targets are detected and the total number of radar scans during the observed time. In Table 4, the detection rates of Target A and B, using the conventional CLEAN detection algorithm, are 45% and 55%, respectively. However, when using the modified CLEAN detection algorithm, the detection rates of Target A and Target B increase to 73% and 87%, respectively. It is clear that the modified CLEAN detection algorithm improves the detection probability.

**Table 4:** Detection rates of two moving targets during the observed time.

Detection Method	Detection Rate	
	Target A	Target B
Conventional CLEAN method	45%	55%
Modified CLEAN method	73%	87%

**Figure 9:**
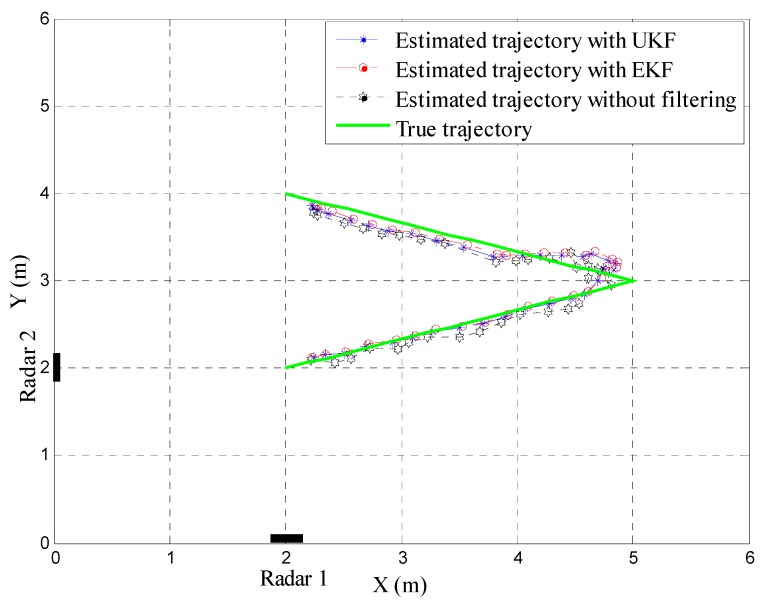
Tracking of a target in two-dimensional coordinates.

The results for tracking with different algorithms are presented in Figure 9 and Table 5. Figure 9 shows the estimated target trajectory with the EKF and UKF and the estimated target trajectory without filtering compared to the true target trajectory. It can be observed from this figure that we can localize and track the target trajectory with two cooperating radars. However, the estimated target trajectory without filtering fluctuates widely around the true trajectory. In particular, the errors are significant when the target moves to the bound of radar range, *i.e.*, the area from 4 m to 6 m along the *x*-axis in Figure 9. After applying the EKF and UKF, the target trajectory is smoothed out, and the estimated trajectory is close to the true target trajectory.

Table 5 summarizes the RMSE comparison results between the estimated target trajectories with the filters and the true target trajectory. The RMSEs of the estimated trajectory with the UKF and EKF and without a filtering method compared to the true trajectory are 0.2260, 0.2373 and 0.2478, respectively. These results verify that the EKF and UKF can improve the precision when estimating the target path. In addition, the UKF provides better performance among the EKF and UKF algorithms.

**Table 5:** RMSE comparison between the estimated target trajectories with filters and the true target trajectory.

Tracking	RMSE (m)
Estimated trajectory without filtering	0.2478
Estimated trajectory by EKF	0.2373
Estimated trajectory by UKF	0.2260

## 4. Conclusions

The detection, localization and tracking of moving targets are important techniques in rescue, emergency and security-related applications. The biggest challenges in IR-UWB radar-signal processing are that the received signals are deteriorated by the target distance, material and shape. The signal perturbation may create large errors when estimating target positions.

In this paper, new signal-processing combinations are suggested for a 2D moving-object tracking system using the IR-UWB radar system. Our developed radar system can enhance the target detection and tracking ratios by using the proposed signal-processing combinations, *i.e.*, the KF-based filtering method used in the clutter reduction step, signal compensation and the 2D jumping-window-based false alarm elimination algorithm used in the detection step and UKF-based target tracking in the tracking step. These techniques are concatenated in the proposed 2D radar system to improve the detection of targets located faraway and to reduce the number of false alarms.

By using two radars as shown in this paper, the target position and trajectory can be determined and tracked in 2D coordinates. However, the detection and tracking can be obviously enhanced by locating more radars in the observation area with the consideration of antenna radiation angles, because the added radars will widen the target detection area.
